# Leukocyte mono-immunoglobulin-like receptor 8 (LMIR8)/CLM-6 is an FcRγ-coupled receptor selectively expressed in mouse tissue plasmacytoid dendritic cells

**DOI:** 10.1038/s41598-018-25646-8

**Published:** 2018-05-29

**Authors:** Ayako Kaitani, Kumi Izawa, Akie Maehara, Masamichi Isobe, Ayako Takamori, Toshihiro Matsukawa, Mariko Takahashi, Yoshinori Yamanishi, Toshihiko Oki, Hiromichi Yamada, Masakazu Nagamine, Shino Uchida, Koichiro Uchida, Tomoaki Ando, Keiko Maeda, Nobuhiro Nakano, Toshiaki Shimizu, Toshiyuki Takai, Hideoki Ogawa, Ko Okumura, Toshio Kitamura, Jiro Kitaura

**Affiliations:** 1 Atopy (Allergy) Research Center, Juntendo University Graduate School of Medicine, 2-1-1 Hongo, Bunkyo-ku, Tokyo 113-8421 Japan; 2 Division of Cellular Therapy/Division of Stem Cell Signaling, The Institute of Medical Science, The University of Tokyo, 4-6-1 Shirokanedai, Minato-ku, Tokyo 108-8639 Japan; 3 Department of Hematology, Hokkaido University Graduate School of Medicine, Sapporo, Hokkaido 060-0808 Japan; 4 Department of Immune Regulation, Graduate School of Medical and Dental Sciences, Tokyo Medical and Dental University, Tokyo, 113-8510 Japan; 5 Department of Pediatrics and Adolescent Medicine, Juntendo University Graduate School of Medicine, 2-1-1 Hongo, Bunkyo-ku, Tokyo 113-8421 Japan; 6 Departments of Gastroenterology Immunology, Juntendo University Graduate School of Medicine, 2-1-1 Hongo, Bunkyo-ku, Tokyo 113-8421 Japan; 7 Department of Experimental Immunology, Institute of Development, Aging, and Cancer, Tohoku University, 4-1 Seiryo, Sendai, 980-8575 Japan

## Abstract

Plasmacytoid dendritic cells (pDCs) produce large amounts of type-I interferon (IFN) in response to viral infection or self nucleic acids. Leukocyte mono-immunoglobulin-like receptor 8 (LMIR8), also called CMRF-35-like molecule-6 (CLM-6), is a putative activating receptor among mouse LMIR/CLM/CD300 members; however, the expression and function of LMIR8 remain unclear. Here, we characterize mouse LMIR8 as a pDC receptor. Analysis of Flag-tagged LMIR8-transduced bone marrow (BM)-derived mast cells demonstrated that LMIR8 can transmit an activating signal by interacting with immunoreceptor tyrosine-based activating motif (ITAM)-containing FcRγ. Flow cytometric analysis using a specific antibody for LMIR8 showed that LMIR8 expression was restricted to mouse pDCs residing in BM, spleen, or lymph node. FcRγ deficiency dampened surface expression of LMIR8 in mouse pDCs. Notably, LMIR8 was detected only in pDCs, irrespective of TLR9 stimulation, suggesting that LMIR8 is a suitable marker for pDCs in mouse tissues; LMIR8 is weakly expressed in Flt3 ligand-induced BM-derived pDCs (BMpDCs). Crosslinking of transduced LMIR8 in BMpDCs with anti-LMIR8 antibody did not induce IFN-α production, but rather suppressed TLR9-mediated production of IFN-α. Taken together, these observations indicate that LMIR8 is an FcRγ-coupled receptor selectively expressed in mouse tissue pDCs, which might suppress pDC activation through the recognition of its ligands.

## Introduction

Paired activating and inhibitory receptor families positively or negatively regulate immune cell activation^1,2^. Examples include CD300, also called leukocyte mono-immunoglobulin-like receptor (LMIR), CMRF-35-like molecule (CLM), and myeloid-associated immunoglobulin-like receptor (MAIR)^3–8^. CD300/LMIR/CLM members harbor highly homologous immunoglobulin-like domains in their extracellular regions; CD300a/LMIR1/CLM-8 and CD300f/LMIR3/CLM-1 are inhibitory receptors that contain the immunoreceptor tyrosine-based inhibitory motif (ITIM) in the cytoplasmic region, while other members are putative activating receptors that are coupled with immunoreceptor tyrosine-based activating motif (ITAM)-bearing adaptor proteins such as FcRγ and DNAX activating protein 12 (DAP12)^3–9^. Lipids or lipid-binding proteins have been identified as ligands for several CD300/LMIR members in mice and humans^9–17^. Accumulated studies using *CD300a*^*−/−*^, *CD300b*^*−/−*^, or *CD300f*^*−/−*^ mice implicate CD300 molecules in the pathogenesis of inflammatory diseases, autoimmune diseases, and infectious diseases^9–12,18–20^.

Plasmacytoid dendritic cells (pDCs) are a unique subset that specializes in the production of type I interferons (IFNs). pDCs recognize viruses and self nucleic acids through Toll-like receptor 7 (TLR7) and TLR9, which are located in endosomal compartments, resulting in the secretion of proinflammatory cytokines and chemokines, via the myeloid differentiation primary response protein 88 (MYD88)-nuclear factor-κB (NF-κB) pathway, and type I interferons (IFNs), via the MYD88-interferon regulatory factor 7 (IRF7) pathways. pDCs can also function as antigen-presenting cells. Accordingly, pDCs participate not only in anti-viral innate immunity but also in adaptive immunity involving autoimmunity^21–25^. Surface markers of pDCs in mice include CD11c, B220, Ly-6C, bone marrow (BM) stromal antigen 2 (BST2), and sialic acid-binding immunoglobulin-like lectin H (Siglec-H)^21–24^. Human pDCs selectively express blood dendritic cell antigen-2 (BDCA2) and immunoglobulin-like transcript 7 (ILT7)^21–24^. Cell surface receptors expressed by pDCs are known to regulate the amplitude of type I IFN production. Notably, high avidity crosslinking of pDC receptors (e.g., BDCA2, ILT7, and NKp44 in humans and Siglec-H and BST2 in mice), interacting with FcRγ or DAP12, attenuates TLR7/9-mediated production of IFN-α or proinflammatory cytokines^21–33^. However, the relevant molecular mechanisms remain unclear.

In the present study, we analyzed the expression and function of mouse LMIR8/CLM-6, demonstrating that LMIR8, an FcRγ-coupled receptor, is selectively expressed in pDCs. In addition, we found that LMIR8 engagement induces cytokine production of BM-derived mast cells (BMMCs) transduced with LMIR8, while it suppresses the TLR9-mediated production of IFN-α in Flt3 ligand-induced BM-derived pDCs (BMpDCs) transduced with LMIR8. Although expression and function of human CD300a/CD300c in pDCs were previously reported^34,35^, this is the first demonstration of a possible specialized role of LMIR8 in mouse pDCs.

## Results

### Mouse LMIR8/CLM-6 is an N-glycosylated surface receptor that is likely expressed in hematopoietic cells

**Figure 1:**
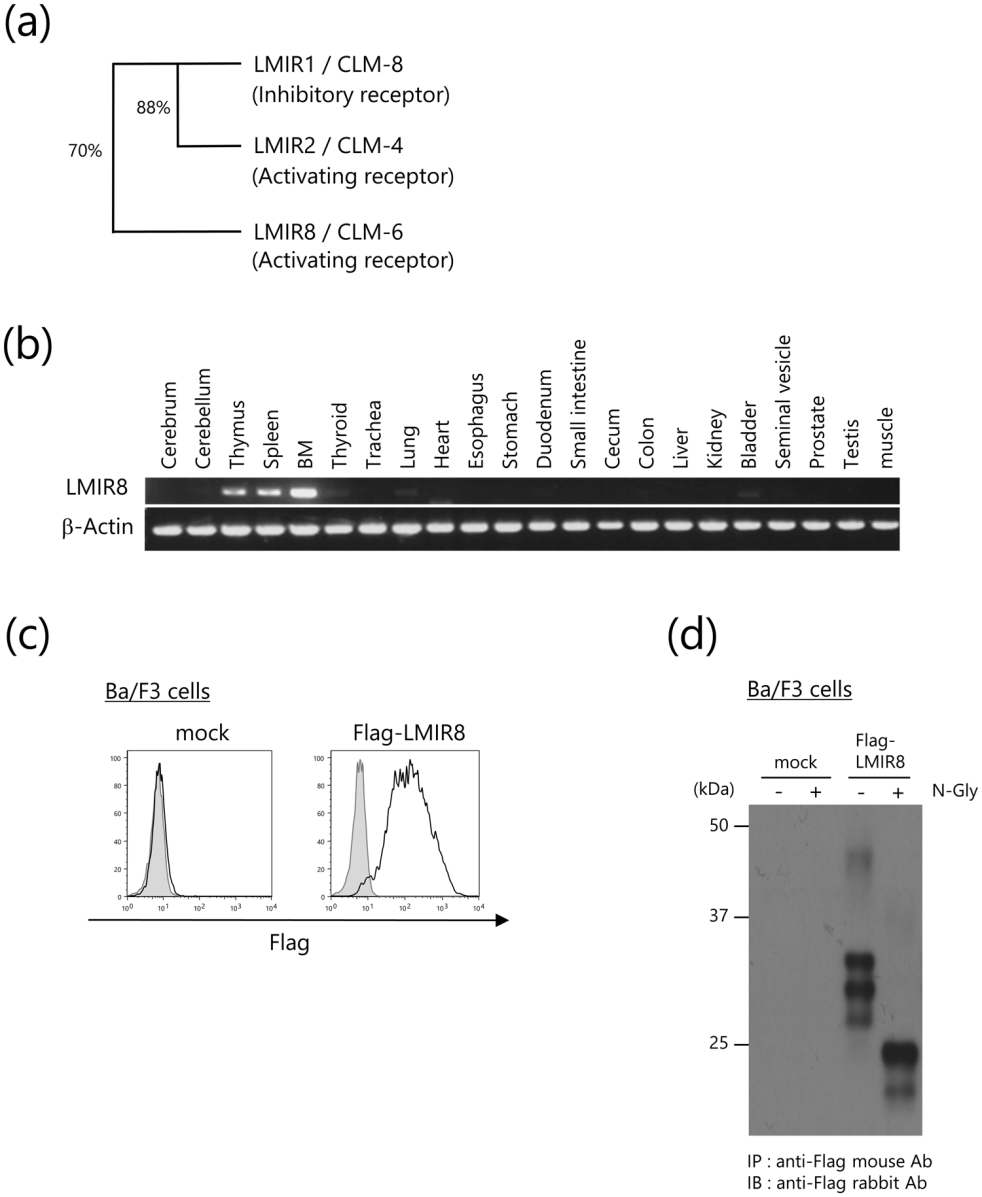
Mouse LMIR8 is an N-glycosylated surface receptor. (**a**) The phylogenetic tree of LMIR1/CLM-8, LMIR2/CLM-4, and LMIR8/CLM-6 is shown based on homology with the immunoglobulin-like domain. Percentages of identity in amino acid sequences of the immunoglobulin-like domain are depicted. (**b**) RT-PCR analysis of LMIR8 or β-actin (control) expression in murine tissues is indicated. (**c**) Ba/F3 cells transduced with Flag-tagged LMIR8 or mock were stained with mouse anti-Flag Ab or a control Ab followed by PE-conjugated anti-mouse IgG goat F(ab’)_2_ Ab. (**d**) Lysates of Ba/F3 cells transduced with either Flag-tagged LMIR8 or mock were immunoprecipitated with mouse anti-Flag Ab. The precipitates treated with or without μM N-glycosidase F were immunoblotted with rabbit anti-Flag Ab. (**b**–**d**) One representative of three independent experiments is shown.

We cloned a full-length cDNA for LMIR8/CLM-6 from a C57BL/6 J mouse BM cDNA library. LMIR8 protein is composed of an N-terminal signal peptide, an extracellular region, a transmembrane domain with a positively charged residue lysine, and a short cytoplasmic tail without signaling motifs such as ITAM and ITIM. LMIR8 contains an immunoglobulin-like domain in the extracellular region that shares 70% identity of amino acid sequences with that of the inhibitory receptor LMIR1 (CLM-8/CD300a) (Fig. 1a)^3–5^. The existence of a positively charged residue lysine in the transmembrane domain of LMIR8 led us to postulate that like other activating LMIR members, LMIR8 might interact with an adaptor protein bearing a negatively charged residue in the transmembrane domain. We then examined expression profiles of LMIR8 in mouse tissues. Reverse transcription polymerase chain reaction (RT-PCR) analysis showed that LMIR8 expression was detectable in BM, spleen, or thymus (Fig. 1b and Supplementary Fig. S1), suggesting that LMIR8 is expressed in hematopoietic cells. Accordingly, we transduced Flag-tagged LMIR8 or mock into the pro-B cell line Ba/F3. Staining of these transfectants with anti-Flag antibody (Ab) displayed surface expression of Flag-tagged LMIR8 in the transduced Ba/F3 cells, but not in mock transfectants, confirming that LMIR8 is a surface receptor (Fig. 1c). As revealed by Western blot analysis using anti-Flag Ab, Flag-tagged LMIR8 protein expression in Ba/F3 cell transfectants was detected as several protein bands (44–46, 29–30, 27–28, or 26 kDa), whereas the same protein was recognized as two bands with lower molecular weights (23–24 or 21 kDa) by pre-treatment with N-Glycosidase F (Fig. 1d and Supplementary Fig. S1). These results indicate that LMIR8 is an N-glycosylated protein, which was supported by the existence of a putative N-glycosylation site within the extracellular domain of LMIR8. Thus, LMIR8 is an N-glycosylated surface receptor that is likely expressed in hematopoietic cells.

### LMIR8 transmits an activating signal in transduced BMMCs

**Figure 2:**
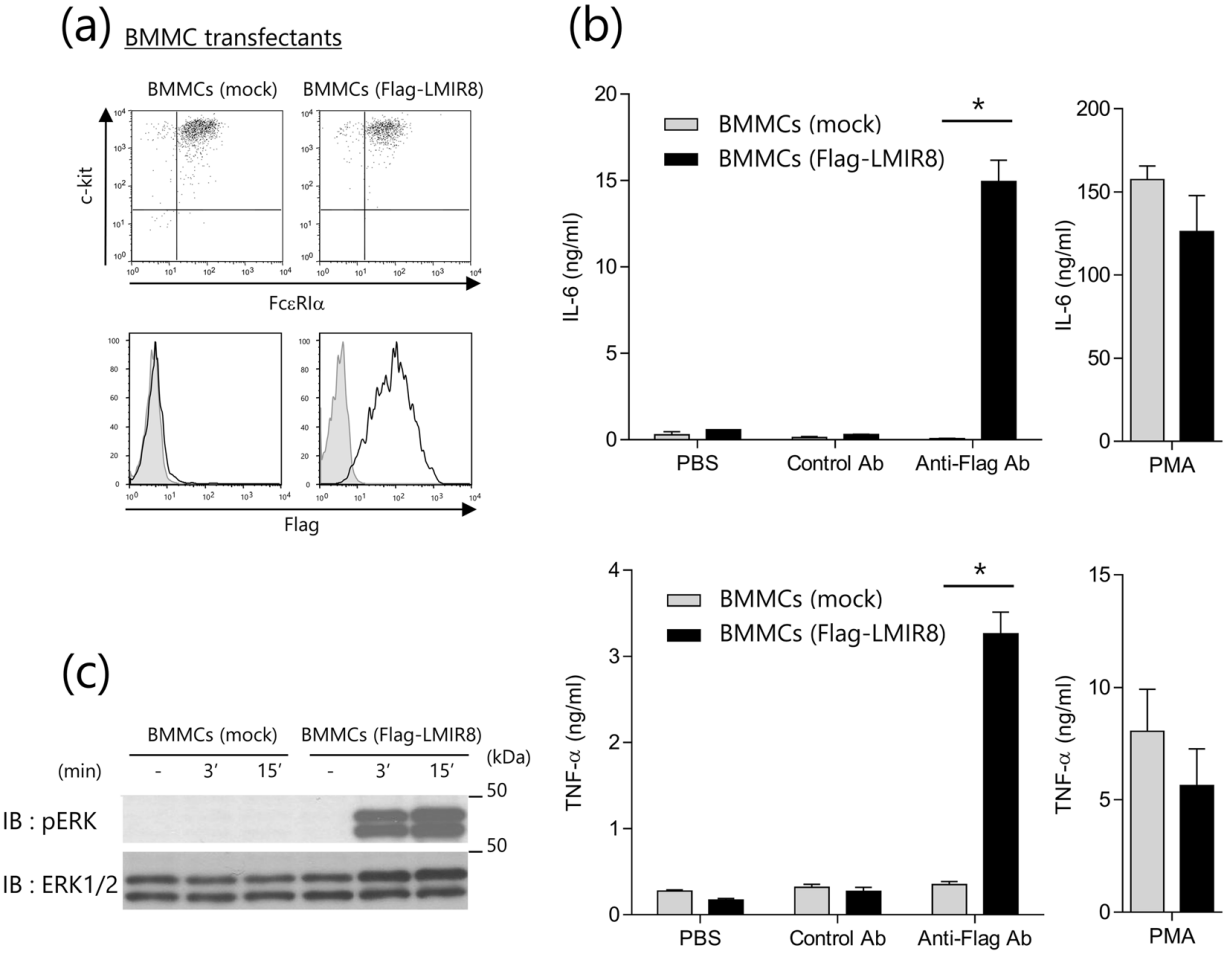
LMIR8 transmits an activating signal in transduced BMMCs. (**a**) BMMCs transduced with Flag-tagged LMIR8 or mock were stained with FITC-conjugated anti-FcεRIα Ab and PE-conjugated anti-c-Kit Ab or with mouse anti-Flag Ab or a control Ab followed by PE-conjugated anti-mouse IgG goat F(ab’)_2_ Ab. (**b**) BMMCs transduced with Flag-tagged LMIR8 or mock were stimulated with plate-coated anti-Flag Ab or a control Ab or with 100 nM PMA or PBS as a control. IL-6 or TNF-α released into the culture supernatants were measured by ELISA. (**c**) BMMCs transduced with Flag-tagged LMIR8 or mock were stimulated with anti-LMIR8 Ab for 3 or 15 min. Cell lysates were subjected to immunoblotting with anti-phospho-p44/42 MAPK (pERK1/2) Ab or anti-ERK1/2 Ab. (**a**,**c**) One representative of three independent experiments is shown. (**b**) All data points correspond to the mean ± S.D. of three independent experiments. Statistically significant differences are shown. *p < 0.01 (Student’s *t*-test).

To test if LMIR8 acts as an activating receptor, Flag-tagged LMIR8 or mock was transduced into BMMCs that express ITAM-containing adaptor proteins such as FcRγ. Equivalent levels of FcεRI and c-Kit were exhibited by these transfectants (Fig. 2a). We confirmed that Flag-tagged LMIR8 was expressed in the transduced BMMCs, but not in mock transfectants (Fig. 2a). When these two BMMC transfectants were stimulated with plate-coated anti-Flag Ab or a control Ab, we found significant cytokine (IL-6 and TNF-α) production of Flag-tagged LMIR8-transduced BMMCs in response to stimulation with plate-coated Flag Ab, but not with a control Ab (Fig. 2b). In contrast, the same stimulation did not induce cytokine production of mock-transduced BMMC (Fig. 2b). Stimulation with phorbol 12-myristate 13-acetate (PMA) led to comparable levels of cytokine production between the two types of BMMC transfectants (Fig. 2b). Thus, LMIR8 engagement induced cytokine production in the transduced BMMC transfectants. Consistent with this, stimulation with anti-Flag Ab caused remarkable activation of extracellular signal-regulated kinase (ERK) in Flag-tagged LMIR8-transduced BMMCs, but not in mock transfectants (Fig. 2c and Supplementary Fig. S2). These results indicate that LMIR8 can act as an activation receptor in the transduced BMMC transfectants.

### FcRγ is required for maintaining maximum surface expression of and transmitting an activation signal by the transduced LMIR8 in BMMC transfectants

**Figure 3:**
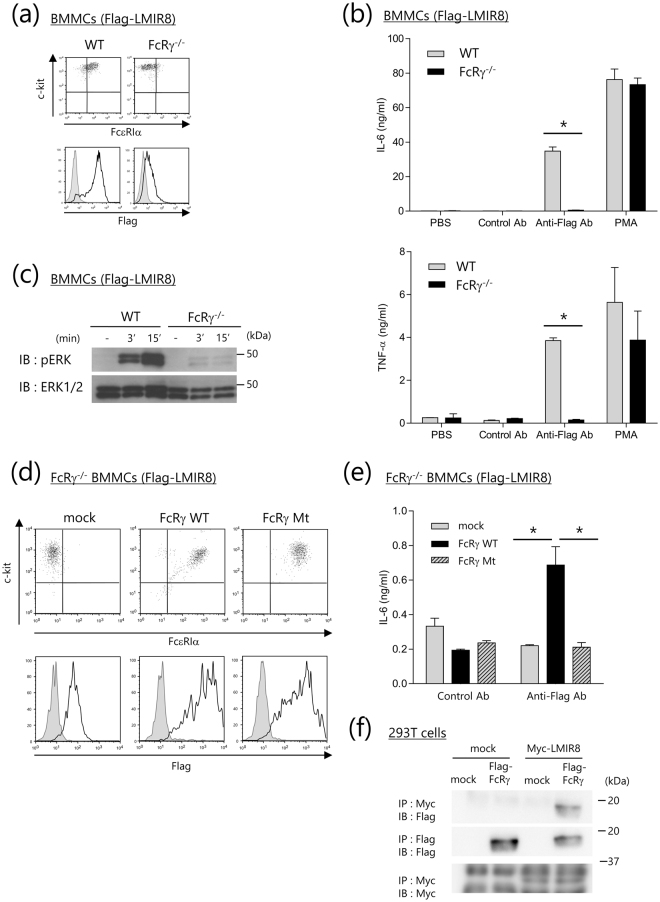
The role of FcRγ in the transduced Flag-taggedLMIR8 in BMMC transfectants. (**a**,**d**) WT or *FcRγ*^*−/−*^ BMMCs transduced with Flag-tagged LMIR8 (**a**) or *FcRγ*^*−/−*^ BMMCs transduced with FcRγ WT, FcRγ Mt, or mock (**d**) were stained with FITC-conjugated anti-FcεRIα Ab and PE-conjugated anti-c-Kit Ab (*upper panel*) or with mouse anti-Flag Ab or a control Ab followed by PE-conjugated anti-mouse IgG goat F(ab’)_2_ Ab (*lower panel*). (**b**,**e**) WT or *FcRγ*^*−/−*^ BMMCs transduced with Flag-tagged LMIR8 (b) or *FcRγ*^*−/−*^ BMMCs transduced with FcRγ WT, FcRγ Mt, or mock (**e**) were stimulated with plate-coated anti-Flag Ab or a control Ab or with 100 nM PMA or PBS as a control. IL-6 or TNF-α released into the culture supernatants were measured by ELISA. (**c**) WT or *FcRγ*^*−/−*^ BMMCs transduced with Flag-tagged LMIR8 were stimulated with anti-Flag Ab for 3 or 15 min. Cell lysates were subjected to immunoblotting with anti-phospho-p44/42 MAPK (pERK1/2) Ab or anti-ERK1/2 Ab. (**f**) Lysates of 293 T cells transiently expressing Myc-tagged LMIR8 or mock together with Flag-tagged FcRγ or mock were immunoprecipitated with anti-Myc Ab or mouse anti-Flag Ab, and then immunoblotted with rabbit anti-Flag Ab or anti-Myc Ab. (**a,c,d,f**) One representative of three independent experiments is shown. (**b,e**) All data points correspond to the mean ± S.D. of three independent experiments. Statistically significant differences are shown. *p < 0.01 (Student’s *t*-test).

To test if FcRγ is an adaptor protein of LMIR8, wild-type (WT) or *FcRγ*^*−/−*^ BMMCs were transduced with Flag-tagged LMIR8. We found that FcRγ deficiency dampened surface expression of FcεRI as expected but did not influence surface expression levels of c-Kit (*upper panels* in Fig. 3a)^6^. Notably, the loss of FcRγ substantially, but not completely, lowered surface expression levels of Flag-tagged LMIR8 in the BMMC transfectants (*lower panels* in Fig. 3a). Possibly related to this, the loss of FcRγ abolished the production of pro-inflammatory cytokines (IL-6 and TNF-α) triggered by Flag-tagged LMIR8 crosslinking with anti-Flag Ab in the BMMC transfectants, presumably in part due to the lowered surface expression of Flag-tagged LMIR8 in the FcRγ-deficient BMMC transfectants (Fig. 3b). We confirmed that FcRγ deficiency did not significantly affect PMA-induced cytokine production of BMMCs transduced with Flag-tagged LMIR8 (Fig. 3b). In addition, FcRγ deficiency dampened Flag-tagged LMIR8-mediated ERK activation of the transduced BMMCs (Fig. 3c and Supplementary Fig. S3). Although it was clear that FcRγ was required for maintaining maximum surface expression of the transduced LMIR8, we asked if FcRγ was indispensable for transmitting an activation signal mediated by the transduced LMIR8 in BMMC transfectants. To this end, *FcRγ*^*−/−*^ BMMCs were transduced with Flag-tagged LMIR8 together with FcRγ WT, FcRγ mutant in which tyrosine residues within the ITAM of FcRγ were replaced with phenylalanine residues, or mock. Notably, *FcRγ*^*−/−*^ BMMCs transduced with FcRγ WT or FcRγ mutant exhibited comparable levels of the transduced Flag-tagged LMIR8 as well as FcεRI on the cell surfaces (Fig. 3d). We confirmed that the loss of FcRγ dampened FcεRI expression and remarkably lowered Flag-tagged LMIR8 expression in the BMMCs transfectants (Fig. 3d). Comparable levels of c-Kit expression were observed in these three transfectants (Fig. 3d). Then, BMMC transfectants were stimulated with plate-coated anti-Flag Ab or a control Ab. The results show that stimulation with anti-Flag Ab, but not with a control Ab, induced significant cytokine production in the transfectants expressing FcRγ WT (Fig. 3e). It should be noted that FcRγ mutant-expressing transfectants did not induce cytokine production at all in response to stimulation with anti-Flag Ab (Fig. 3e). These results indicate that the ITAM of FcRγ was indispensable for delivering an activating signal of LMIR8 in the BMMC transfectants. Co-immunoprecipitation experiments verified that LMIR8 could physically interact with FcRγ in the transiently transfected 293 T cells (Fig. 3f and Supplementary Fig. S3). Collectively, FcRγ was required for maintaining maximum surface expression of and transmitting an activation signal through the transduced LMIR8 in BMMC transfectants.

### LMIR8 is selectively expressed in mouse tissue pDCs

**Figure 4:**
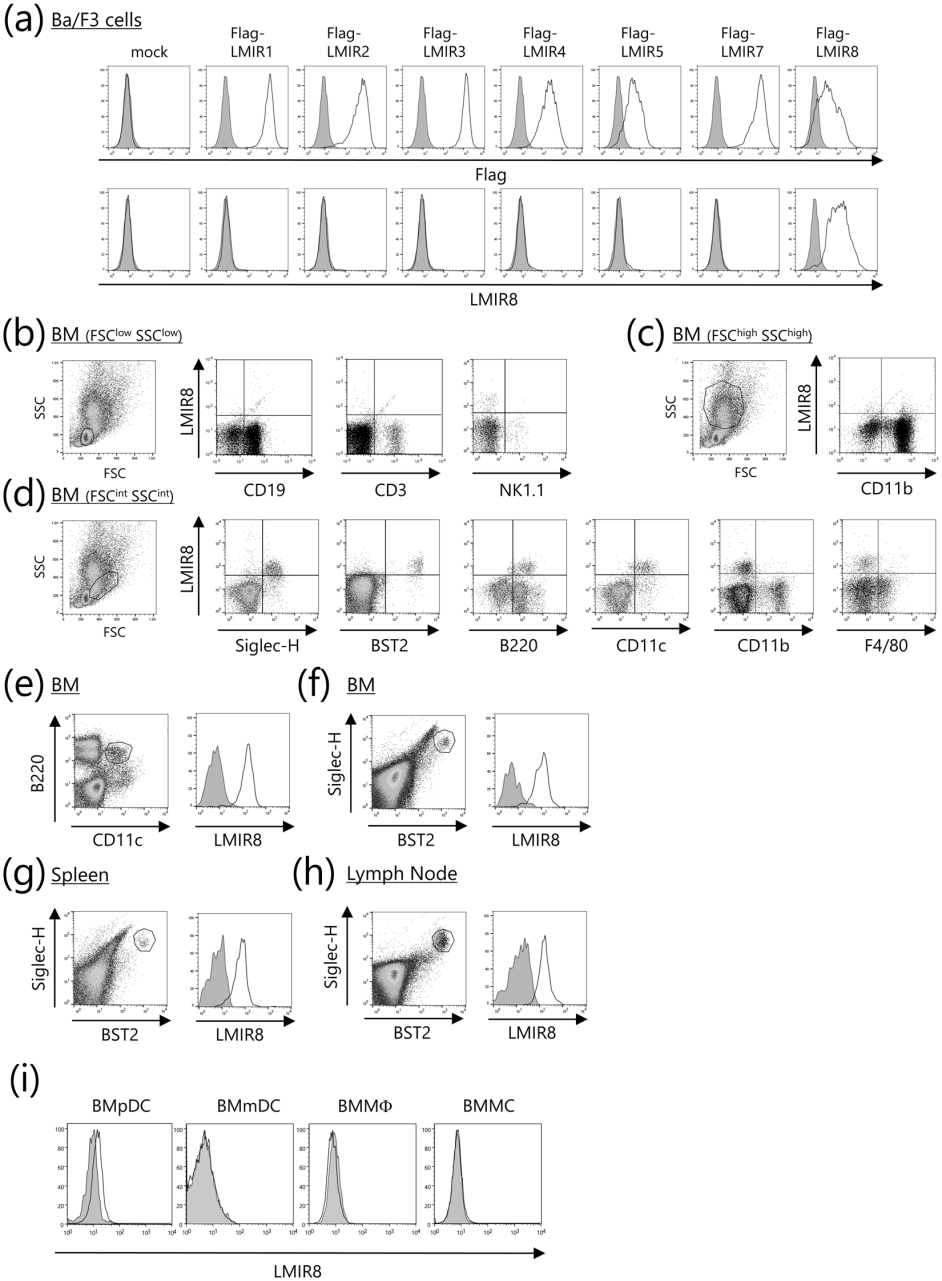
LMIR8 is highly expressed in pDCs. (**a**) Ba/F3 cells transduced with Flag-tagged LMIR1, LMIR2, LMIR3, LMIR4, LMIR5, LMIR7, LMIR8, or mock were stained with mouse anti-Flag Ab or a control Ab followed by PE-conjugated anti-mouse IgG goat F(ab’)_2_ Ab (*upper panel*) or with biotinylated anti-LMIR8 Ab or biotinylated rat IgG2a Ab followed by PE-conjugated streptavidin. (**b–h**) Single cell suspensions were prepared from BM (**b**–**f**), spleen (**g**), or lymph node (**h**). (**b**–**d**) Cells were stained with biotinylated anti-LMIR8 Ab or biotin rat IgG2a Ab followed by PE-conjugated streptavidin and FITC-conjugated Abs as indicated. FSC^low^SSC^low^ populations (**b**), FSC^high^SSC^high^ populations (**c**), or FSC^int^SSC^int^ populations (**d**) were gated. (**e**–**h**), BM cells (**e,f**), spleen cells (**g**), or lymph node cells (**h**) were stained with FITC-conjugated anti-CD11c Ab and APC-conjugated anti-B220 Ab (**e**) or with FITC-conjugated anti-BST2 Ab and APC-conjugated anti-Siglec-H Ab (**f**–**h**), and then with biotinylated anti-LMIR8 Ab or biotinylated rat IgG2a Ab followed by PE-conjugated streptavidin. CD11c^+^B220^+^ (**e**) or BST2^+^Siglec-H^+^ (**f**–**h**) cell populations were gated and analyzed for LMIR8 expression. (**i**) Flt3 ligand-induced BMpDCs, BMmDCs, BMMΦ, or BMMCs were stained with biotinylated anti-LMIR8 Ab or biotinylated rat IgG2a Ab followed by PE-conjugated streptavidin. One representative of four independent experiments is shown.

To analyze the expression profiles of LMIR8 in hematopoietic cells, we generated a monoclonal Ab for LMIR8. Like anti-Flag Ab, this Ab detected surface expression of the transduced Flag-tagged LMIR8 in Ba/F3 cells (Fig. 4a). By contrast, anti-LMIR8 Ab did not recognize Flag-tagged mouse LMIR1 (CLM-8), LMIR2 (CLM-4), LMIR3 (CLM-1), LMIR4 (CLM-5), LMIR5 (CLM-7), LMIR7 (CLM-3), or mock on the surface of the transduced Ba/F3 cells^3–8^, although we found surface expression of each LMIR member tested by using anti-Flag Ab (Fig. 4a). Thus, we confirmed the specificity of a newly generated monoclonal Ab for LMIR8. We then stained BM cells with anti-LMIR8 Ab and performed flow cytometric analysis. When FSC^low^SSC^low^ or FSC^high^SSC^high^ BM cells were gated, we found that LMIR8 were not expressed in CD3^+^ T cells, CD19^+^ B cells, NK1.1^+^ NK cells, or CD11b^+^ myeloid cells (Fig. 4b,c). Notably, LMIR8 was expressed in a subpopulation of FSC^int^SSC^int^ BM cells, which expressed Siglec-H, BST-2, B220, and CD11c (Fig. 4d). In addition, we found that CD11c^+^B220^+^ or BST2^+^Siglec-H^+^ BM cells corresponding to pDCs expressed high levels of LMIR8 (Fig. 4e,f)^21–24^. In addition, LMIR8 was highly expressed in BST2^+^Siglec-H^+^ pDCs in both spleen and lymph node (Fig. 4g,h). It should be noted that LMIR8 was only weakly expressed in Flt3 ligand-induced BMpDCs (Fig. 4i). Neither BM-derived myeloid dendritic cells (BMmDCs), BM-derived macrophages (BMΦ), nor BMMCs expressed LMIR8 (Fig. 4i). Collectively, these results indicate that LMIR8 was selectively expressed in pDCs in mouse tissues.

### FcRγ-coupled LMIR8 is a suitable marker for pDC

**Figure 5:**
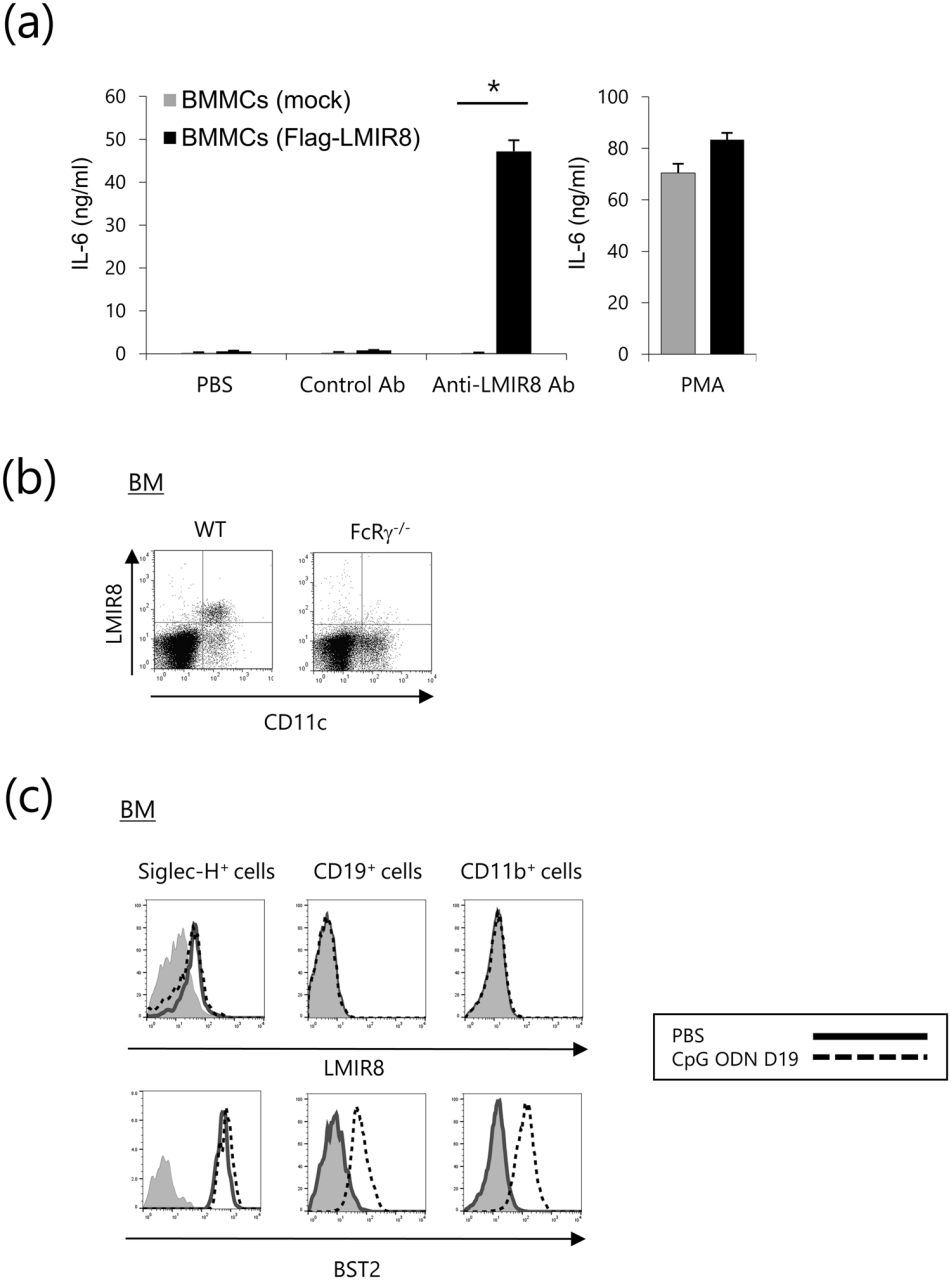
FcRγ-coupled LMIR8 is a suitable marker for PDC. (**a**) BMMCs transduced with Flag-tagged LMIR8 or mock were stimulated with plate-coated anti-LMIR8 Ab or a control Ab or with 100 nM PMA or PBS as a control. IL-6 released into the culture supernatants were measured by ELISA. All data points correspond to the mean ± S.D. of three independent experiments. Statistically significant differences are shown. *p < 0.01 (Student’s *t*-test). (**b**) BM cells from WT or *FcRγ*^−/−^ mice were stained with FITC-conjugated anti-CD11c Ab and biotinylated anti-LMIR8 Ab or biotinylated rat IgG2a Ab followed by PE-conjugated streptavidin. (**c**) BM cells from WT mice before or 24 h after an intravenous injection of 12.5 μg of CpG ODN D19 were stained with either FITC-conjugated anti-Siglec-H Ab, anti-CD19 Ab, or anti-CD11b Ab and biotinylated anti-LMIR8 Ab or biotinylated rat IgG2a Ab followed by PE-conjugated streptavidin. Siglec-H^+^ (*left panel*), CD19^+^ (*middle panel*), or CD11b^+^ (*right panel*) cell populations were gated and analyzed for LMIR8 expression. One representative of four independent experiments is shown.

We then asked if stimulation with this anti-LMIR8 Ab induces LMIR8-dependent cytokine production in transduced BMMCs. The results showed that similar to anti-Flag Ab, plate-coated anti-LMIR8 Ab, but not with control Ab, stimulated IL-6 production at both mRNA and protein levels in Flag-tagged LMIR8-transduced BMMCs (Fig. 5a and Supplementary Fig. S4a). In addition, LMIR8 engagement enhanced lipopolysaccharide (LPS)-stimulated IL-6 production in Flag-tagged LMIR8-transduced BMMCs, indicating that LMIR8 cooperates with TLR4 to upregulate cytokine production in transduced BMMCs (Supplementary Fig. S4b). Thus, LMIR8 can transmit an activating signal in transduced BMMCs. To test if FcRγ is an adaptor protein for LMIR8 in pDC, WT or *FcRγ*^*−/−*^ BM cells were stained with anti-LMIR8 Ab, demonstrating that the loss of FcRγ dampened surface expression of LMIR8 in BM pDCs (Fig. 5b). Thus, FcRγ was indispensable for surface expression of LMIR8 in pDC. We next asked if stimulation with a TLR9 agonist affected surface expression of LMIR8 in pDCs. *In vivo* administration of a TLR9 agonist, CpG oligodeoxynucleotide (ODN) D19, induced expression of BST2, a surface marker for PDC in steady state, in CD19^+^ B cells or CD11b^+^ myeloid cells in BM, although we found no expression of BST2 in these cell populations before stimulation (Fig. 5c)^36^. By contrast, neither CD19^+^ B cells nor CD11b^+^ myeloid cells in BM expressed detectable levels of LMIR8 irrespective of *in vivo* administration of a TLR9 agonist (Fig. 5c). The same stimulation slightly upregulated expression of BST-2, but not of LMIR8, in Siglec-H^+^ pDCs in BM (Fig. 5c). These results indicate that LMIR8 is a suitable marker for pDC in tissues.

### LMIR8 engagement with anti-LMIR8 Ab inhibits the TLR9-mediated IFN-α production in transduced pDCs

**Figure 6:**
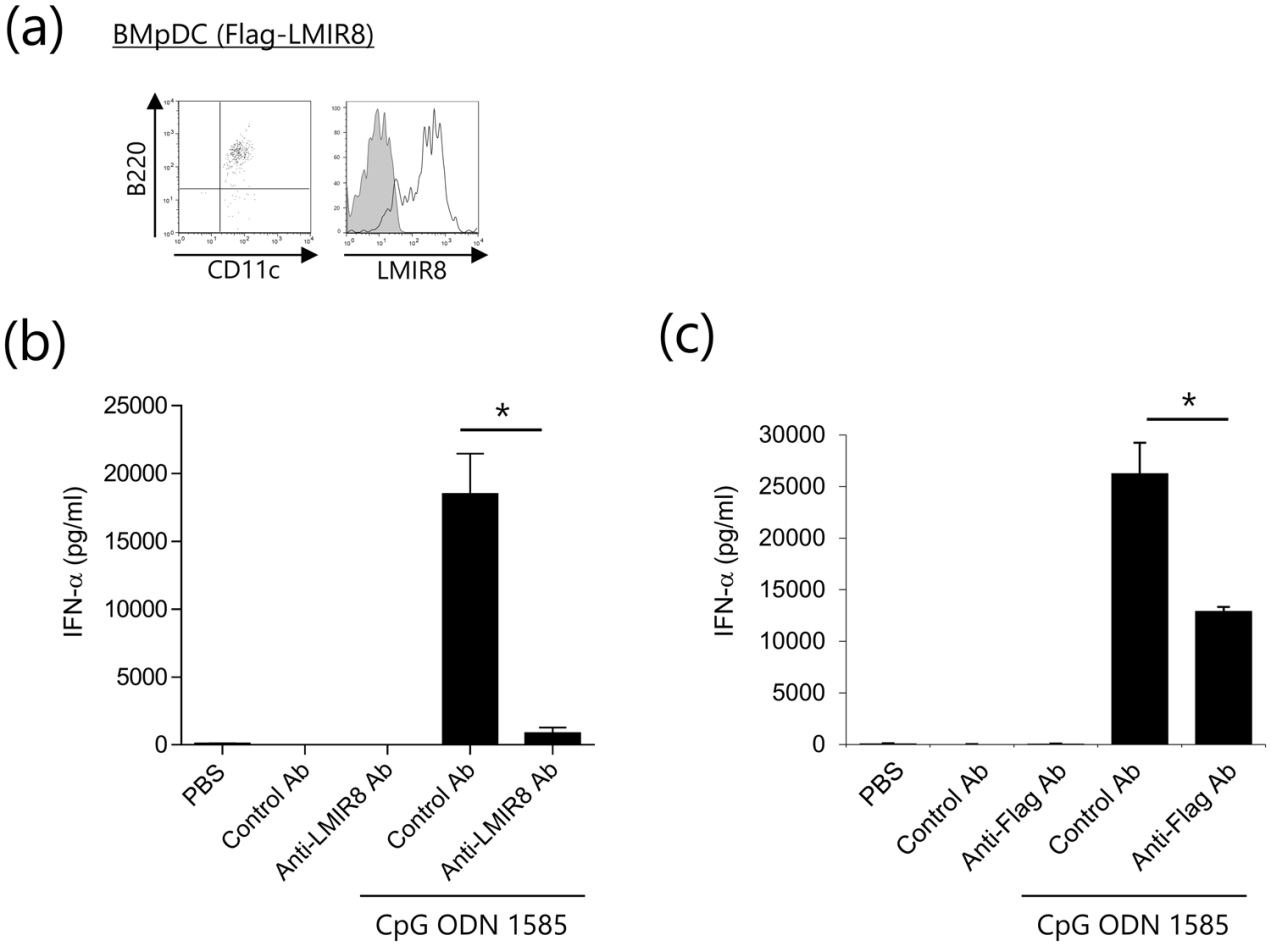
LMIR8 engagement with anti-LMIR8 Ab inhibits the TLR9-mediated cytokine production in transduced pDCs. (**a**) WT BMpDCs transduced with Flag-tagged LMIR8 or mock were stained with FITC-conjugated anti-CD11c Ab and APC-conjugated anti-B220 Ab or with biotinylated anti-LMIR8 Ab or biotinylated rat IgG2a Ab followed by PE-conjugated streptavidin. One representative of three independent experiments is shown. (**b**,**c**) Flag-tagged LMIR8-transduced WT BMpDCs were stimulated with plate-coated anti-LMIR8 Ab or a control Ab (**b**) or with anti-Flag Ab or a control Ab (**c**) in the presence or absence of 5 μM CpG ODN 1585 or PBS as a control. IFN-α released into the culture supernatants were measured by ELISA. All data points correspond to the mean ± S.D. of three independent experiments. Statistically significant differences are shown. *p < 0.01 (Student’s *t*-test).

Then, we examined the effect of LMIR8 engagement with anti-LMIR8 Ab on the activation of pDCs. Since BMpDCs expressed lower levels of LMIR8 than did pDC in tissues, BMpDCs were transduced with Flag-tagged LMIR8 or mock. Staining of these transfectants with anti-LMIR8 Ab displayed high levels of LMIR8 in Flag-tagged LMIT8-transduced BMpDCs (Fig. 6a). Neither plate-coated anti-LMIR8 Ab nor a control Ab induced significant levels of IFN-α in Flag-tagged LMIT8-transduced BMpDCs (Fig. 6b). By contrast, stimulation with a TLR9 agonist, CpG ODN 1585, induced significant production of IFN-α; however, we found that co-stimulation with anti-LMIR8 Ab, but not with a control Ab, substantially decreased IFN-α production (Fig. 6b). Similarly, co-stimulation with anti-Flag Ab decreased TLR9-meditaed IFN-α production (Fig. 6c). These results indicated that LMIR8 signals inhibit TLR9-mediated IFN-α production in transduced pDCs. However, similar co-stimulation with anti-LMIR8 Ab failed to do so in Flt3 ligand-induced BMpDCs or in pDCs sorted from BM or spleen, which expressed lower levels of LMIR8 than did the LMIR8-transduced pDCs. In any case, the signals triggered by LMIR8 engagement downregulated TLR9-mediated pDC activation.

### Ceramide, sphingomyelin, phosphatidylserine, and phosphatidylethanolamine are not ligands for LMIR8

**Figure 7:**
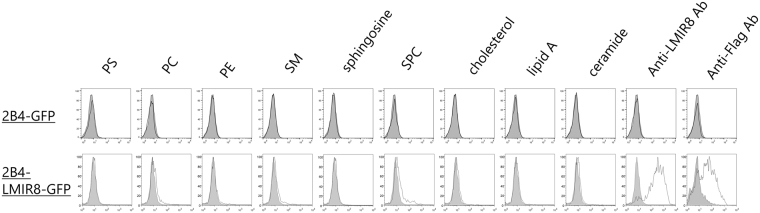
Ceramide, sphingomyelin, phosphatidylserine, and phosphatidylethanolamine are not ligands for LMIR8. Flow cytometry of GFP expression of 2B4-LMIR8-GFP cells or 2B4-GFP cells that were incubated for 24 h on plates coated with indicated lipids or with anti-LMIR8 Ab. PC, PS, PE, SM, or SPC indicates phosphatidylcholine, phosphatidylserine, phosphatidylethanolamine, sphingomyelin, or sphingosylphosphocholine. One representative of three independent experiments is shown.

To identify LMIR8 ligands, we performed functional reporter assays^9^. We transduced a chimera receptor, in which the extracellular domain of LMIR8 was fused to the transmembrane domain of LMIR3 followed by an intracellular domain of human CD3ζ containing an ITAM, into the parental reporter cells 2B4-GFP, where GFP expression would be induced by the activation of nuclear factor of activated T-cells (NFAT)^9,37,38^, to generate the new reporter cells LMIR8-2B4-GFP. If an extracellular domain of the chimera receptor were engaged by LMIR8 ligands, GFP expression would be induced in LMIR8-2B4-GFP cells. We confirmed that plate-coated anti-LMIR8 Ab induced GFP expression in LMIR8-2B4-GFP cells, but not in 2B4-GFP cells (Fig. 7). We then asked if lipids, including ceramide, sphingomyelin, phosphatidylserine, or phosphatidylethanolamine, which are ligands for several LMIR/CD300 members, acted as ligands for LMIR8^9–17^. However, GFP expression was not induced by any plate-coated lipids tested (Fig. 7). The ligands for LMIR8 remain to be identified.

## Discussion

In the present study, we generated a specific Ab for LMIR8 and demonstrated that LMIR8 is exclusively expressed in CD11c^+^B220^+^Siglec-H^+^BST2^+^ pDCs in mouse tissues. In accordance with a previous report showing that BST2 is expressed in pDCs and plasma cells in steady state, while its expression is induced in many cell types following IFN-inducing stimulation^36^, our results confirm that BST2 is unsuitable as a PDC marker, in particular under inflammatory conditions. In addition, given that Siglec-H is expressed not only in pDCs but also in specialized macrophage subsets in the spleen, lymph nodes, or brain^39,40^, LMIR8 may be a more suitable marker for pDC; LMIR8 expression in cell types other than pDCs was neither detected nor induced under the inflammatory conditions tested. Unlike Siglec-H expression which is down-regulated upon activation^41^, LMIR8 expression remained significantly uninfluenced by stimulation with a TLR9 agonist in pDCs. However, it should be noted that Flt3 ligand-induced BMpDCs expressed extremely lower levels of LMIR8 than did pDCs in tissues. Accordingly, LMIR8 or Siglec-H appeared to be expressed in pDCs in different stages of differentiation^21,22^. In any case, we provide evidence that LMIR8 is a novel and suitable marker for mouse pDCs in tissues. Since the transcription factor E2-2 binds to a large proportion of pDC-enriched genes^22,42^, it is possible that E2-2 also regulates expression of LMIR8. Crossing mice expressing Cre recombinase under the control of the LMIR8 promoter with mice carrying the floxed target gene might make it possible to analyze the protein of interest expressed in pDCs *in vivo*.

On the basis of our results regarding LMIR8-transduced BMMCs, it is plausible to assume that LMIR8 functions as an activating receptor in the BMMC transfectants; FcRγ is required for both maintaining maximum surface expression of and delivering activating signals through the transduced LMIR8. Consistent with this, FcRγ was indispensable for surface expression of endogenous LMIR8 in mouse pDCs. However, this is in sharp contrast to the finding that FcRγ is not required for the surface expression of FcRγ-coupled LMIR7 in mast cells and macrophages^8^. It is important to note that LMIR8 engagement inhibited the production of IFN-α in LMIR8-transduced BMpDCs in response to a TLR9 agonist, which supports previous studies pointing to several surface receptors that interact with ITAM-containing adaptor proteins as a negative regulator of TLR7/9 signaling; engagement of FcRγ-coupled BDCA2 or ILT7 or DAP12-coupled NKp44 in human pDCs or of DAP12-coupled Siglec-H in mouse pDCs attenuates TLR7/9-mediated activation of pDCs^21–33^. Although we do not know the relevant mechanisms, different localization of internalized FcRγ-coupled LMIR8 bound by anti-LMIR8 Ab and TLR9 in endosomes might explain this phenomenon^22–24^. In addition, a recent finding that the CD2-associated adaptor protein (CD2AP)/SH2 domain-containing inositol phosphatase 1 (SHIP1) complex positively regulates BDCA2 and/or FcRγ signaling in humans pDCs to inhibit the E3 ubiquitin ligase Cbl^22,23,43^ led us to speculate that the similar regulation of signaling might be involved in FcRγ-coupled LMIR8-mediated inhibition of TLR9 signaling in mice. Because human pDCs have significant surface expression of CD300a, but not of other CD300 members including CD300c^15,35^, a previous report showing that engagement of human CD300a and CD300c with their cross-linking antibody reduced TNF-α production and increased IFN-α production in human pDCs might reflect the function of human CD300a^34^. This indicates that the regulation of pDC activation by CD300 members is different between mice and humans. Moreover, we need to examine the effect of LMIR8 engagement with physiological ligands, which remain to be identified, on TLR7/9 signaling in mouse pDCs to fully understand the role of LMIR8 in pDC activation. Because ceramide and sphingolipids have been identified as ligands for several CD300/LMIR members^9–17^, it is possible that LMIR8, a pDC receptor, recognizes specific viral components, including lipids, which directly regulate immune responses to certain viral infections. Further studies to analyze LMIR8-deficient mice in viral infection models or in autoimmune disease models as well as to identify ligands for LMIR8 will further our understanding of the physiological functions of LMIR8 in mouse pDCs to develop therapeutic strategies against pDCs-associated diseases.

In conclusion, LMIR8 is an FcRγ-coupled receptor that is selectively expressed in pDCs. Our results suggest that LMIR8 signals might negatively regulate the activation of pDCs in response to viral infections or to self nucleic acids, implicating LMIR8 in innate immunity or autoimmunity.

## Methods

### Antibodies and Other Reagents

Rat anti-LMIR8 monoclonal IgG2a Ab was generated by ACTGen, Inc. Mouse anti-Flag IgG1 Ab (M2), rabbit anti-Flag Ab, and mouse IgG1 Ab (MOPC21) were purchased from Sigma-Aldrich. Mouse anti-Myc Ab (9E10) was from Roche Diagnostics. R-phycoerythrin (PE)-conjugated anti-c-Kit, fluorescein isothiocyanate (FITC)-conjugated anti-FcεRIα, CD3, CD19, CD11b, Siglec-H, BST2, B220, CD11c, NK1.1, or F4/80 antibodies, PE-Cy7-conjugated anti-B220 Ab, and peridinin chlorophyll protein complex (PerCP)-conjugated anti-Siglec-H Ab were from BioLegend. PE-conjugated anti-mouse IgG goat F(ab’)_2_ Ab was from Beckman Coulter. Rat IgG_2a_ Ab and PE-conjugated streptavidin were from eBioscience. Anti-ERK 1/2 Ab was from Santa Cruz Biotechnology. Anti-phospho-p44/42 MAPK (EER1/2) was from Cell Signaling Technology. Anti-LMIR8 Ab or rat IgG2a Ab was biotinylated with sulfo-NHS-LC-biotin (Pierce, Thermo Fisher Scientific) according to the manufacturer’s instructions. Cytokines were obtained from R&D Systems. Peptide-N-Glycosidase F (PNGase F) was from New England Biolabs. CpG oligodeoxynucleotide (ODN) 1585 or D19 (Hokkaido System Science Co.) was used for *in vitro* or *in vivo* experiments, respectively. Sphingosine, sphingomyelin (SM), and sphingosylphosphorylcholine (SPC) were from BIOMOL; C-24 ceramide was from Toronto Research Chemicals, Inc.; lipid A, lysophosphatidylcholine (lysolecithin), and cholesterol were from Avanti Polar Lipids, Inc.; 1,2-Dipalmitoyl-sn-glycero-3-phosphocholine (PC), 1,2-Dipalmitoyl-sn-glycero-3-phosphoserine (PS), and 1,2-Dipalmitoyl-sn-glycero-3-phosphoethanolamine (PE) were from Echelon Biosciences Inc. All other reagents were from Sigma-Aldrich unless stated otherwise.

### Mice and cells

Cell lines Ba/F3, 2B4-GFP (a kind gift from Takashi Saito, RIKEN Research Center for Allergy and Immunology, Yokohama, Japan), and HEK 293 T were used^6,9,37,38^. C57BL/6 J WT and *FcRγ*^*−/−*^ mice^44^ were used at 8–10 weeks of age. Mouse tissues were removed from C57BL/6 J mice for RNA extraction. BM, spleen, and lymph node cells were purified from mice and used as previously described^6–8^. All procedures were approved by an institutional review committee of the University of Tokyo and Juntendo University. BMMCs were generated from BM cells in the presence of 10 ng/mL IL-3 as described. BMΦ, BMmDCs, and BMpDCs were generated from BM cells in the presence of 10 ng/mL M-CSF, 20 ng/mL GM-CSF, and 50 ng/mL Flt3-ligand, respectively, as described^6–8,45^.

### Ethics statement

All animal experiments were approved by the ethical committee of the University of Tokyo (approval no 20–8) and Juntendo University (approval no 290108). All the methods were carried out in accordance with the approved guidelines and regulations.

### Gene Expression Analysis

Expression of LMIR8 was analyzed by reverse transcriptase-polymerase chain reaction (RT-PCR) as previously described^6–8^. Total RNAs were extracted from each tissue or transduced BMMCs with TRIzol reagents (Invitrogen) and reverse-transcribed by using High Capacity cDNA Reverse Transcription Kits (Applied Biosystems). A fragment of LMIR8 was amplified with primers 5′-TTCAGATATGCATGGAGGCCATT-3′ and 5′-TGATACCGTTCCCAGGGCGT-3′. For normalization, a fragment of β-actin was amplified with 5′-CATCACTATTGGCAACGAGC-3′ and 5′-ACGCAGCTCAGTAACAGTCC-3′. Relative expression levels of mouse IL-6 among samples were measured by real-time RT-PCR as described^8^. The following primers were used: 5′-GCCAGAGTCCTTCAGAGAGATACA-3′ (forward) and 5′-CTTGGTCCTTAGCCACTCCTTC-3′ (reverse) for IL-6 and 5′-GAAGTGTGACGTTGACATCC-3′ (forward) and 5′-GTACTTGCGCTCAGGAGGAG-3′ (reverse) for β-actin. Relative gene expression levels were calculated using standard curves generated by serial dilutions of cDNA and normalized to β-actin expression levels. Product quality was checked by melting curve analysis via LightCycler software (Roche Diagnostics).

### Plasmid Constructs

On the basis of the sequence data, the cDNAs of mouse LMIR8 (GenBank^TM^ accession number XM_017314637.1) and FcRγ (GenBank^TM^ accession number NM_010185) were isolated by PCR from a BM cDNA library derived from C57BL/6J mice and their sequences were confirmed. A cDNA fragment of LMIR8 lacking the signal sequence was tagged with a Flag or Myc epitope at the N-terminus to generate Flag- or Myc-tagged LMIR8. A signaling lymphocyte-activating molecule (SLAM) signal sequence (a gift from Hisashi Arase, Osaka University, Osaka, Japan)^46^ and Flag- or Myc-tagged LMIR8 were subcloned into a pMXs-IRES-puro^r^ (pMXs-IP)^47^ retroviral vector to generate pMXs-FLAG- or Myc-LMIR8-IP. A cDNA fragment of FcRγ lacking the signal sequence was tagged with a Flag epitope at the N-terminus to generate Flag-tagged FcRγ. A SLAM signal sequence and Flag-tagged FcRγ were subcloned into a pMXs-IRES-blasticidin (pMXs-IB) to generate pMXs-Flag-FcRγ-IB^6–8,47^. To generate the Flag-tagged FcRγ (Y82F-Y93F) mutant (Flag-FcRγ-Mt), two-step PCR mutagenesis was performed by using pMXs-Flag-FcRγ-IB as a template. A SLAM signal sequence and Flag-tagged FcRγ-Mt were subcloned into pMXs-IB to generate pMXs-Flag-FcRγ-Mt-IB. To generate a chimera receptor LMIR8-CD3ζ, SLAM signal sequence-Flag-LMIR8, excluding transmembrane and intracellular domains, was fused to the transmembrane domain of LMIR3 and an intracellular domain of human CD3ζ (Naoki Matsumoto, University of Tokyo, Tokyo, Japan). LMIR8-CD3ζ was subcloned into pMXs-IP to generate pMXs-Flag-LMIR8-CD3ζ-IP^9,14,15^. All constructs were verified by DNA sequencing.

### Transfection and Infection

Retroviral transfection was performed as previously described^47,48^. Briefly, retroviruses were generated by transient transfection of PLAT-E packaging cells. Cells were infected with retroviruses in the presence of 10 μg/mL Polybrene and selected with puromycin and/or blasticidin.

### Flow Cytometry

Flow cytometric analysis of the stained cells was performed with a FACSCalibur (BD Biosciences) equipped with CellQuest software and FlowJo software (Tree Star) as previously described^6–9^. For detection of LMIR8, cells were incubated with 10 μg/mL biotinylated anti-LMIR8 Ab or biotinylated rat IgG2a Ab before incubation with PE-conjugated streptavidin. For detection of Flag (Flag-tagged receptor), cells were incubated with 10 μg/mL anti-Flag Ab or mouse IgG1 Ab before incubation with PE-conjugated anti-mouse IgG goat F(ab’)_2_ Ab.

### Cell stimulation and measurement of cytokines

Plates were coated overnight with 20 μg/mL of each Ab. BMMCs transfectants expressing Flag-tagged LMIR8 or mock were stimulated with plate-coated mouse ant-Flag Ab or mouse IgG1 as a control, plate-coated rat anti-LMIR8 Ab or rat IgG2a as a control with or without 1000 ng/ml LPS, or with 100 nM PMA for 24 h. BMpDCs were stimulated with 5 mM CpG ODN 1585 for 24 h on plate-coated rat anti-LMIR8 Ab or rat IgG2a as a control. The concentrations of cytokines/chemokines in the supernatants were measured using enzyme-linked immunosorbent (ELISA) kits for IL-6 and TNF-α (R&D Systems) or using the Procarta cytokine assay kit (Affymetrix) for IFN-α. Alternatively, lipids dissolved in methanol (10 μg/mL) or methanol only as a control were added to plates and air-dried for the reporter assay^9^. 2B4-GFP or LMIR8-2B4-GFP cells were cultured for 24 h on plates coated with the indicated lipids or vehicle or with anti-LMIR8 Ab.

### Biochemistry

BMMCs expressing FLAG-tagged LMIR8 or mock were stimulated by plate-coated anti-Flag Ab or mouse IgG_1_ Ab as a control, for the indicated time. Equal amounts of cell lysates were immunoblotted with anti-phospho-ERK1/2 Ab or anti-ERK1/2 Ab. Equal amounts of HEK 293 T cells transiently expressing Myc-tagged LMIR8 or mock together with Flag-tagged FcRγ or mock were immunoprecipitated with anti-Myc Ab, and were immunoblotted with rabbit anti-Flag Ab or anti-Myc Ab. Alternatively, the same cell lysates were immunoprecipitated with mouse anti-Flag Ab, and were immunoblotted with rabbit anti-Flag Ab. Immunoprecipitation and Western blotting were done as previously described^6–8^.

### Statistical Analysis

Data are shown as the mean ± S.D., and statistical significance was determined by Student’s **t** test with *p < 0.01 taken as statistically significant.

## Electronic supplementary material

Supplemental information
