# Supplementary material for: Leukocyte mono-immunoglobulin-like receptor 8 (LMIR8)/CLM-6 is an FcRγ-coupled receptor selectively expressed in mouse tissue plasmacytoid dendritic cells
Source: Sci Rep. 2018 May 29;8:8259. doi: 10.1038/s41598-018-25646-8 (PMC5974347; doi:10.1038/s41598-018-25646-8)

Leukocyte mono-immunoglobulin-like receptor 8 (LMIR8)/CLM-6 is an FcRγ-coupled receptor selectively expressed in mouse tissue plasmacytoid dendritic cells

Ayako Kaitani[1,2], Kumi Izawa[1,2], Akie Maehara[1,2], Masamichi Isobe[1,2], Ayako Takamori[1], Toshihiro Matsukawa[2,3], Mariko Takahashi[2], Yoshinori Yamanishi[2,4], Toshihiko Oki[2], Hiromichi Yamada[1,5], Masakazu Nagamine[1], Shino Uchida[1,6], Koichiro Uchida[1], Tomoaki Ando[1], Keiko Maeda[1], Nobuhiro Nakano[1], Toshiaki Shimizu[1,5], Toshiyuki Takai[7], Hideoki Ogawa[1], Ko Okumura[1], Toshio Kitamura[2], and Jiro Kitaura[1,2]

[1]Atopy (Allergy) Research Center, Juntendo University Graduate School of Medicine, 2-1-1 Hongo, Bunkyo-ku, Tokyo 113-8421, Japan.

[2]Division of Cellular Therapy/Division of Stem Cell Signaling, The Institute of Medical Science, The University of Tokyo, 4-6-1 Shirokanedai, Minato-ku, Tokyo 108-8639, Japan.

[3]Department of Hematology, Hokkaido University Graduate School of Medicine, Sapporo, Hokkaido 060-0808, Japan.

[4]Department of Immune Regulation, Graduate School of Medical and Dental Sciences, Tokyo Medical and Dental University, Tokyo 113-8510, Japan.

[5]Department of Pediatrics and Adolescent Medicine, Juntendo University Graduate School of Medicine, 2-1-1 Hongo, Bunkyo-ku, Tokyo 113-8421, Japan.

[6]Departments of Gastroenterology Immunology, Juntendo University Graduate School of Medicine, 2-1-1 Hongo, Bunkyo-ku, Tokyo 113-8421, Japan.

[7]Department of Experimental Immunology, Institute of Development, Aging, and Cancer, Tohoku University, 4-1 Seiryo, Sendai 980-8575, Japan

Corresponding author:

Toshio Kitamura, M.D. Ph.D., Division of Cellular Therapy, Advanced Clinical Research Center, The Institute of Medical Science, The University of Tokyo, 4-6-1 Shirokanedai, Minato-ku, Tokyo 108-8639, Japan; Phone: (+81-3) 5449-5759; Fax: (+81-3) 5449-5428; email: kitamura@ims.u-tokyo.ac.jp and

Jiro Kitaura. M.D. Ph.D., Atopy Research Center, Juntendo University Graduate School of Medicine, 2-1-1 Hongo, Bunkyo-ku, Tokyo 113-8421, Japan; Phone (+81-3) 5802-1591; Fax: (+81-3) 3813-5512; email: j-kitaura@juntendo.ac.jp

Supplementary Figure legends

Supplementary Figure 1. Full-length gel and blot images for Figure 1b and Figure 1d.

Supplementary Figure 2. Full-length blot images for Figure 2c.

Supplementary Figure 3. Full-length blot images for Figure 3c and Figure 3f.

Supplementary Figure 4. LMIR8 can transmit an activating signal in transduced BMMCs.

(a) BMMCs transduced with Flag-tagged LMIR8 were stimulated with plate-coated anti-LMIR8 Ab or a control Ab or PBS as a control for 1 h. Transcript levels of IL-6 quantified by real-time RT-PCR analysis in BMMCs transduced with Flag-tagged LMIR8. Data are expressed. (**b**) BMMCs transduced with Flag-tagged LMIR8 were stimulated with plate-coated anti-LMIR8 Ab or a control Ab with or without 1000 ng/ml LPS for 24 h. IL-6 released into the culture supernatants were measured by ELISA. All data points correspond to the mean ± S.D. of three independent experiments. Statistically significant differences are shown. Statistically significant differences are shown. *$p < 0.01$ (Student's *t*-test).

Supplementary Figure 1: Full-length gel and blot images for Figures 1

(Figure 1b)

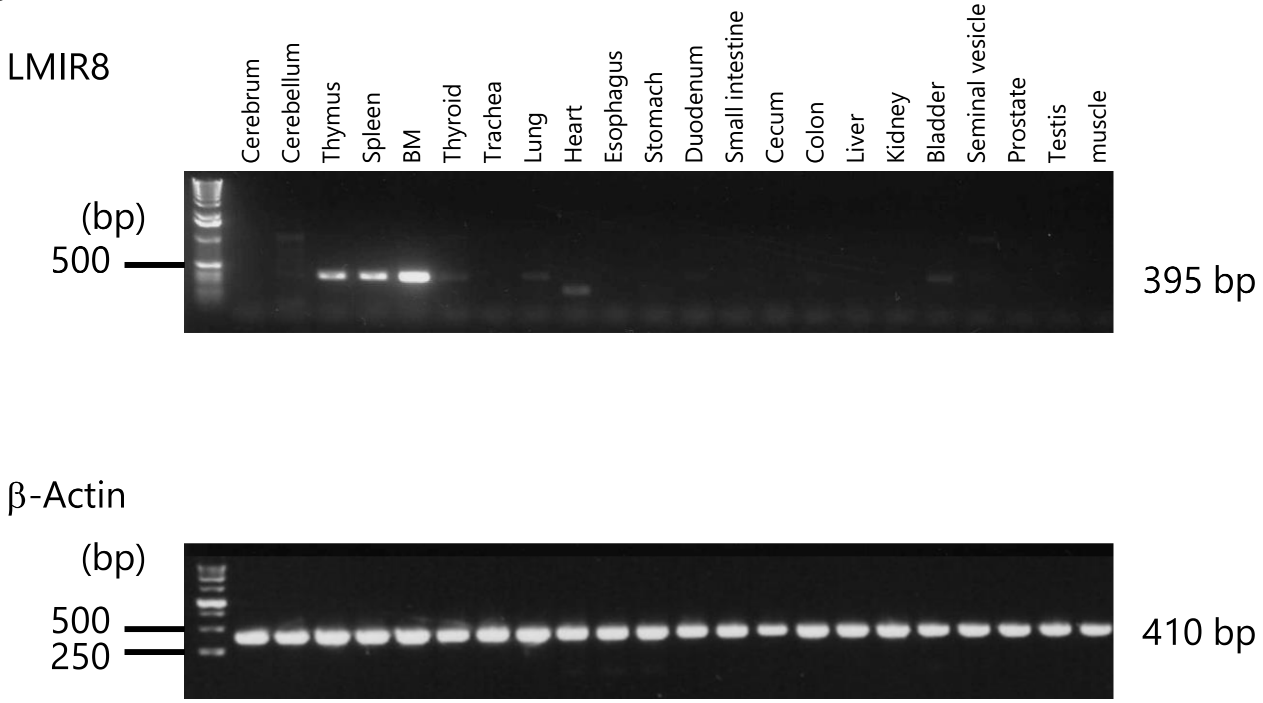

(Figure 1d)

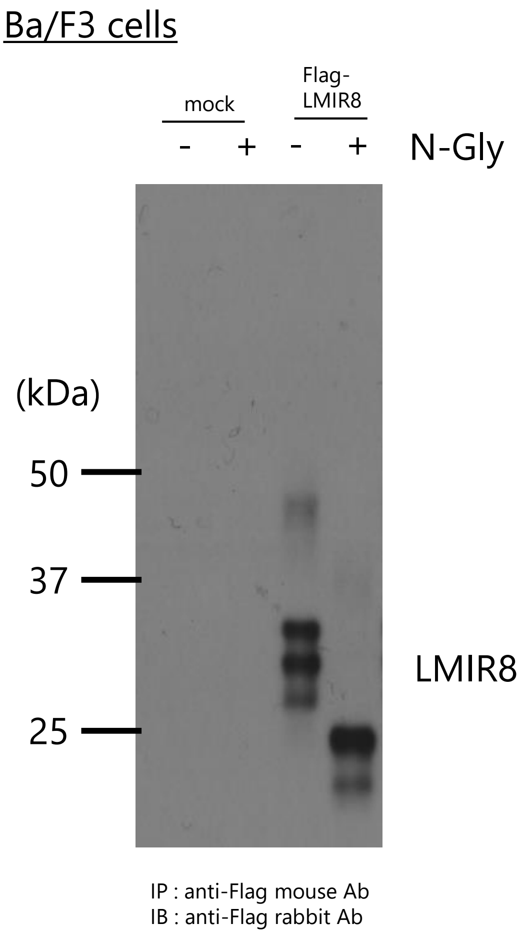

Supplementary Figure S2: Full-length blot images for Figures 2

(Figure 2c)

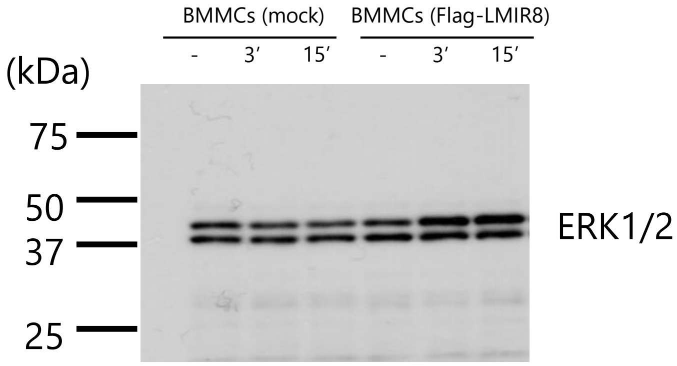

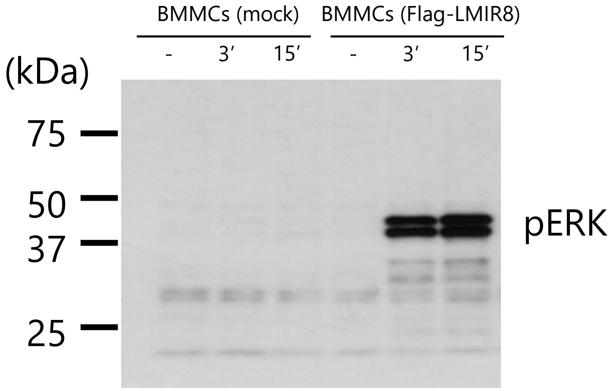

Supplementary Figure S3: Full-length blot images for Figures 3

(Figure 3c)

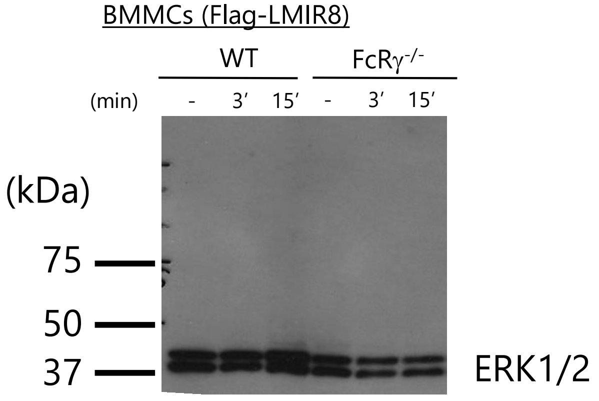

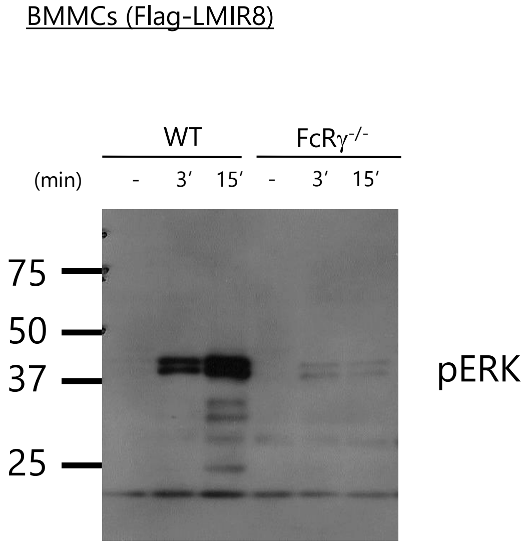

(Figure 3f)

293T cells

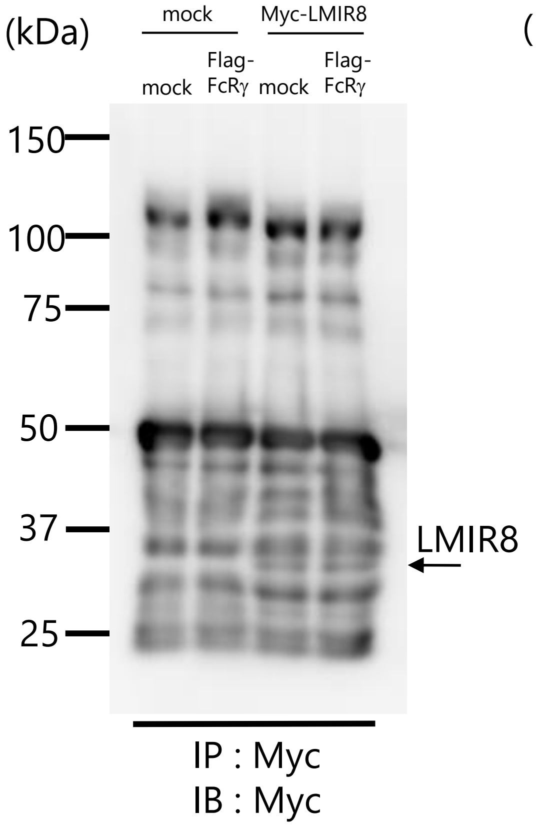

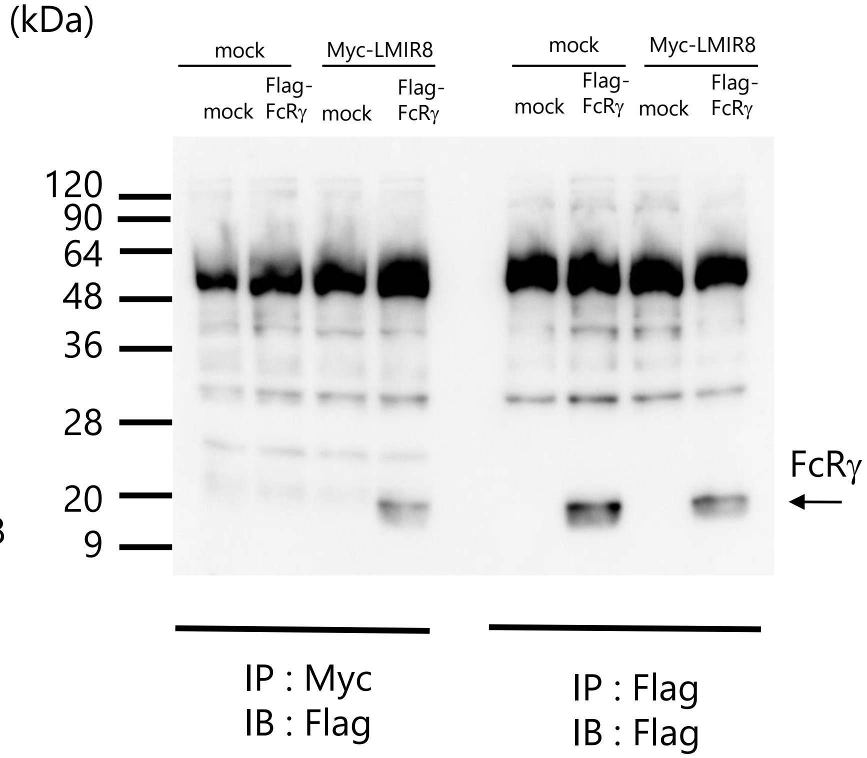

Supplementary Figure S4

(a)

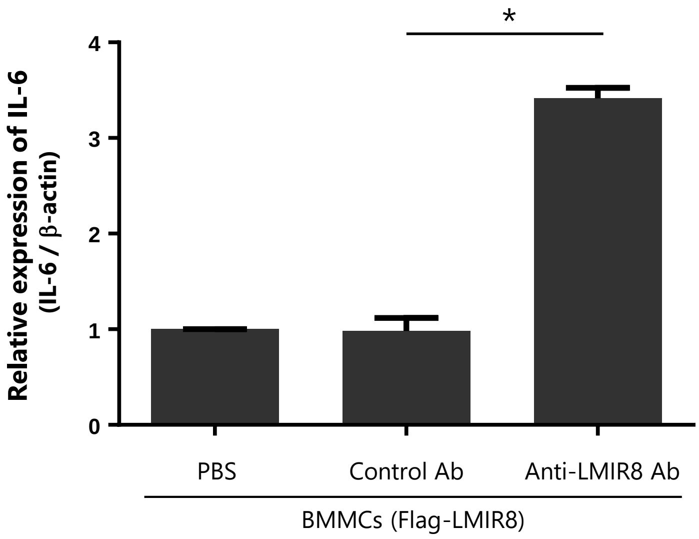

(b)

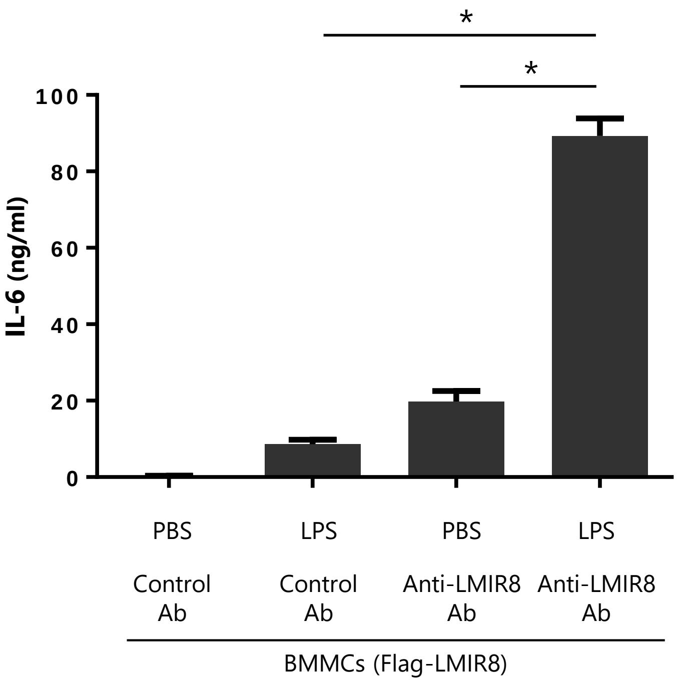

Supplement: Supplementary file 1 — Supplemental information [file 41598_2018_25646_MOESM1_ESM.pdf]
